# Processing, Microstructure, and Performance of Robocast Clay-Based Ceramics Incorporating Hollow Alumina Microspheres

**DOI:** 10.3390/ma17071603

**Published:** 2024-03-31

**Authors:** Yanfang Wu, Junjie Lan, Mingxuan Wu, Mingjun Wu, Li Tian, Hui Yang, Qijiang Li, Yue Li

**Affiliations:** 1 School of Materials Science and Engineering, Zhejiang University, Hangzhou 310058, China; 11926036@zju.edu.cn (Y.W.); yanghui@zju.edu.cn (H.Y.); 2 Chinese Celadon Institute, Lishui University, Lishui 323000, China; wumingxuan2023@gmail.com; 3 Wenzhou Research Institute, Zhejiang University, Wenzhou 325006, China; lanjj1127@126.com; 4 Longquan Celadon Museum, Longquan 323700, China; wumingjunlq@163.com (M.W.); emmett323700@gmail.com (L.T.); 5 Research Center of Ancient Ceramic, Jingdezhen Ceramic University, Jingdezhen 333001, China

**Keywords:** 3D printing, non-contact restoration, hollow Al_2_O_3_ microspheres, Longquan celadon, lightweight reinforcement

## Abstract

The restoration of ancient ceramics has attracted widespread attention as it can reveal the overall appearance of ancient ceramics as well as the original information and artistic charm of cultural relics. However, traditional manual restoration is constrained due to its time-consuming nature and susceptibility to damaging ancient ceramics. Herein, a three-dimensional (3D) printing technique was employed to accurately restore Chinese Yuan Dynasty Longquan celadon using hollow Al_2_O_3_ microsphere-modified 3D printing paste. The results show that the hollow Al_2_O_3_ microsphere content plays a vital role in the printability, physical properties, and firing performance of the modified 3D printing paste. The printed green bodies show no noticeable spacing or voids under moderate rheological conditions. The as-prepared ceramic body modified with 6 wt.% hollow Al_2_O_3_ microspheres and fired at 1280 °C exhibits optimal bending strength of 56.66 MPa and a relatively low density of 2.16 g∙cm^−3^, as well as a relatively uniform longitudinal elastic modulus and hardness along the interlayer. This 3D printing technique based on hollow Al_2_O_3_ microsphere-modified paste presents a promising pathway for achieving non-contact and damage-free restoration of cultural relics.

## 1. Introduction

Numerous ancient ceramics of diverse styles have been discovered at archeological sites and are presently showcased in museums worldwide. Longquan celadon, a collection of green-glazed Chinese ceramics, holds a significant historical position, boasting a lineage spanning over 1700 years [1]. Its production reached its peak during the Yuan Dynasty (A.D. 1271–1368), leading to the discovery of numerous Longquan celadon kiln sites and cultural artifacts during archaeological excavations. However, the restoration of these celadon cultural artifacts has evolved into a laborious and intricate task [2,3].

Currently, two primary categories of restoration methods for ancient ceramics exist: manual methods and non-contact methods. Manual methods entail repairing ancient ceramics predominately by hand through manual comparison, mold casting, sampling, and bonding. However, this technique often leads to extended production cycles and collateral damage due to the repeated handling of ancient ceramics. In contrast, non-contact restoration methods, primarily involving 3D scanning, reverse modeling, and 3D printing, offer shorter restoration periods and mitigate the deterioration of cultural artifacts caused by repeated handling [4]. Compared with manual restoration techniques, 3D printing emerges as a viable and effective method for restoring a diverse range of ceramic artifacts, encompassing diverse dimensions and shapes [5,6].

Various 3D printing techniques have been implemented in the construction of complex ceramic structures, such as selective laser melting/sintering (SLM/SLS) [7], binder jetting [8], robocasting or direct ink writing (DIW) [9,10,11], the fused deposition modeling of ceramics (FDM/FDC) [12], ink-jetting [13], stereolithography (SLA), and digital light processing (DLP) [14]. Among these techniques, classic FDM and DIW are extrusion printing methods preferred for fabrication due to their convenience. The FDM technique mainly prints a filament of thermoplastic printing material [15,16], which requires filament formation. Although FDM has been adapted for the production of ceramic parts by blending thermoplastic binder material with ceramic powder, difficulties arise from the need to remove the polymeric binder component from the printed part before sintering, a process that can potentially lead to cracks and blisters in the final sintered ceramic parts, a challenge shared with other thermoplastic shaping processes for ceramics, such as injection molding, thermoplastic extrusion, and pressing [17,18]. The DIW technique, suitable for various materials, such as ceramics, composite materials, and hydrogels, offers advantages including a wide range of applicable raw materials, high molding accuracy, and flexible manufacturing. DIW is deemed a more suitable 3D printing technique for the non-contact restoration of ancient ceramics [9,19,20]. Importantly, during the DIW 3D printing processes, the rheological behavior and mechanical properties of restoration materials play a crucial role in producing high-performance restoration parts [21,22,23,24].

At present, the restoration materials utilized in 3D printing-based restoration are primarily polymers [25], plaster [26], and inorganic material [27]. Compared with inorganic material, other materials are prone to deformation, aging, and the development of defects over prolonged storage periods, making them less suitable for the long-term preservation of ancient ceramics [28]. In general, restoration materials for robocasting should possess low density and high strength to avoid compression damage to ceramic fragments. Hence, it is essential to utilize lightweight robocasting materials while maintaining high mechanical properties [29]. Previous studies suggest that designing a light cellular structure or introducing a pore-forming agent could aid in achieving light structural control [30,31]. Additionally, alumina (Al_2_O_3_), known for its favorable physical and mechanical properties and being a primary component in Longquan celadon, serves as an ideal filler for Longquan celadon restoration [32]. Notably, hollow alumina microspheres, with their hollow structure, large specific surface area, and high chemical stability, serve as important modified materials to reduce the density and enhance the strength of the ceramic body [33]. However, to the best of our knowledge, limited research has been conducted on the influence of hollow Al_2_O_3_ microspheres on the microstructure and properties of 3D printing paste materials, particularly in preparing lightweight reinforced 3D-printed ceramics for ancient ceramic restoration applications. These research gaps underscore the necessity for further exploration and development in this field.

In this work, a non-contact restoration method for Chinese Yuan Dynasty Longquan celadon utilizing the DIW 3D printing technique was demonstrated. By incorporating hollow Al_2_O_3_ microspheres (Al_2_O_3_ HMs), the rheological properties and printability of the 3D printing paste were optimized, along with enhancing the mechanical properties, volume density, and microstructure of the fired ceramics. Based on 3D laser scanning and reverse modeling, the 3D printing paste material, with high strength, low density, and excellent printability, was further applied to the non-contact restoration of Chinese Yuan Dynasty Longquan celadon as a case study.

## 2. Materials and Methods

### 2.1. Materials

Chinese Yuan Dynasty Longquan celadon pottery (code: LQYD008) was provided by Longquan Celadon Museum, Wuhan, China. Huangtan clay, Zhuxiang clay, Zijinclay, and porcelain powder were purchased from Zhejiang Tianfeng Ceramics Co., Ltd., Hangzhou, China. Potassium feldspar was purchased from Sibelco India Minerals Pvt., Ltd., Hyderabad, India. Hollow Al_2_O_3_ microspheres (Al_2_O_3_ HM) were purchased from Zhengzhou Yuli Industrial Co., Ltd., Zhengzhou, China. Hydroxypropyl methylcellulose (HPMC) with a viscosity of 100,000 mPa∙s was purchased from Shanghai Aladdin Biochemical Technology Co., Ltd., Shanghai, China.

### 2.2. Preparation of Al_2_O_3_ HM-Modified Pastes

Firstly, a pseudo-classic clay was prepared using the ball milling method, with its composition matching that of Longquan celadon pottery (LQYD008) (see Figure S1 and Tables S1–S4) (The cited references provided in the Supplementary Material). Subsequently, the as-prepared pseudo-classic clay, weighing between 1700 and 2000 g, was thoroughly mixed with an aqueous solution of 0.4 g HPMC in 860 g H_2_O. Different mass fractions of Al_2_O_3_ HM (see Figure S2) were then gradually added to the clay mixture according to the formulations listed in Table 1. The combined weight of clay and Al_2_O_3_ HM was kept constant at 2000 g. After stirring for 30 min, uniformly mixed pastes with mass fractions of 0 wt.%, 3 wt.%, 6 wt.%, 9 wt.%, 12 wt.%, and 15 wt.% were prepared, respectively, denoted as S-0, S-1, S-2, S-3, S-4, and S-5, respectively.

### 2.3. 3D Printing of Al_2_O_3_ HM-Modified Pastes

**Figure 1:**
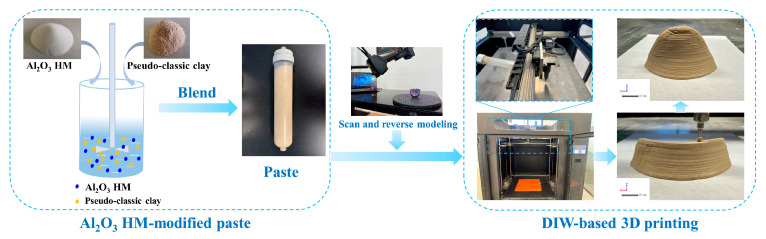
Schematic illustration of 3D printing sequence of Al_2_O_3_ HM-modified ceramic pastes.

Utilizing 3D scanning and reverse modeling, a custom-built DIW 3D printer was employed to prepare the printed samples and Longquan celadon fragmentary part using Al_2_O_3_ HM-modified pastes. In this process, the as-prepared pastes were loaded into the printing barrel and extruded to conform to the predetermined shape following the pre-programmed printing route. The chosen printing parameters included a nozzle diameter of 0.86 mm, a printing speed of 50 mm/s, and an extrusion pressure ranging from 0.35 MPa to 0.4 MPa (see Figure 1).

### 2.4. Firing Process of 3D-Printed Green Body

**Figure 2:**
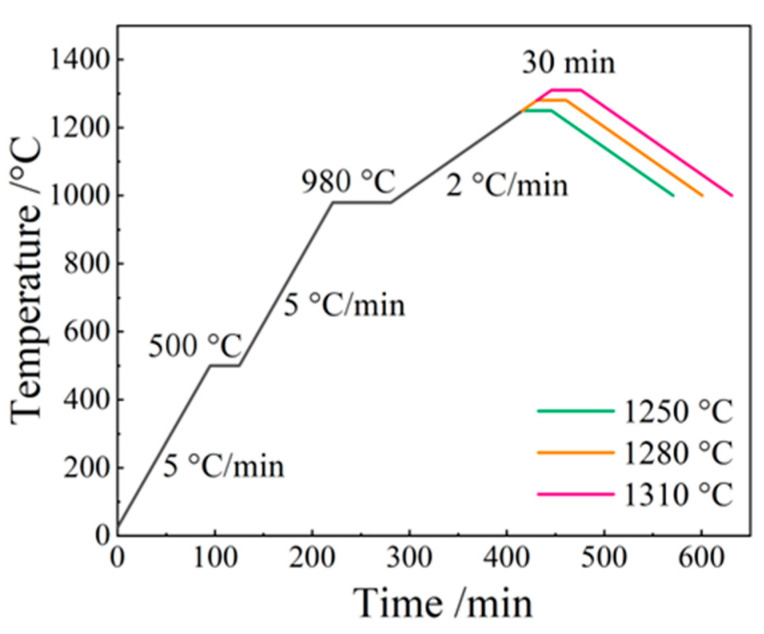
The temperature–time curve of the 3D printing samples.

After printing, all 3D-printed green body samples were covered with plastic wrap and left to air dry for 24 h [34]. Subsequently, the samples were fired according to the temperature–time curve shown in Figure 2. The heating process involved increases of 5 °C/min from room temperature to 500 °C, with a holding temperature at 500 °C maintained for 0.5 h. This was followed by additional increases of 5 °C/min from 500 °C to 980 °C, with a holding temperature maintained at 980 °C for 1 h. The heating process then continued at a rate of 2 °C/min from 1050 °C to various temperatures ranging from 1250 °C to 1310 °C, and with each firing temperature held for 0.5 h. Finally, the samples underwent a slow cooling process within the furnace.

### 2.5. Material Characterization

The rheological behavior of the 3D printing pastes containing different amounts of Al_2_O_3_ HM was characterized using a shear rate and shear stress rheometer (Anton Paar, Graz, Austria). The steady-state viscosity as a function of shear rate was measured from about 0.01 to 10 s^−1^ at 25 °C using a four-bladed vane with dimensions of 10 mm in diameter and 20 mm in length, within a cylindrical cup with an inner diameter of 30 mm.

Bending strength tests were carried out using a strength tester (Instron, High Wycombe, Wycombe, UK). Prior to testing, the samples were dried at 110 °C ± 5 °C for 8 h. The distance between the supporting knife edges of the strength tester was set to 50 mm, and the dried sample was positioned on the supporting knife edge. A loading rate of 40 N/s was applied and the maximum load recorded upon fracture. The width and thickness of the fracture were measured using a micrometer. The bending strength of the samples was calculated using Equation (1):

(1) $$\sigma_{f}=\frac{3FL}{2bh^{2}}$$

where $\sigma_{f}$ represents the bending strength, F is the fracture load of the specimens, L is the distance of the supporting knife edge, and b and h are the width and depth of the specimen fracture, respectively.

Thermostability was evaluated using a water-cooling method. Fired 3D-printed samples were heated to 180 °C for 30 min in an oven. After 10 min, the samples were immersed into a container filled with 20 °C water for 5 min, and then, observed for surface cracks.

Nanoindentation tests were conducted to determine the elastic modulus (E) and hardness (H) using a nanoindenter (Bruker, MI, USA) with basic QS trapezoid loading equipped with a three-sided pyramidal diamond tip (Berkovich tip with a radius of about 100 nm). A peak load of 1000 mN was applied to investigate the indentation hardness and Young’s modulus as a function of penetration depth. During the test, the indenter was driven into the sample surface under a load that gradually increased until the prescribed peak load was reached, and then, the indenter was unloaded gradually after being held at peak load for 5 s. Each sample underwent three indentations at each prescribed peak load level. The mass loss and density of the modified samples were tested and calculated using Equations (S1) and (S2).

A scanning electron microscope (CarlZeissAG, Oberkonchen, Germany) mounted with an energy-dispersive spectroscope (CarlZeissAG, Oberkochen, Germany) was used to analyze the as-prepared samples. X-ray diffraction (XRD) was conducted using a D8 Advance X-ray diffractometer (Bruker, Saarbrücken, Germany), with Cu Kα radiation (λ = 1.54 Å), collecting data between 2*θ* = 5° and 90° with a step size of 0.02°. A laser particle size analyzer (Sympatec GmbH, Clausthal-Zellerfeld, Germany) was used to analyze the particle size distribution. ImageJ 1.53k software was utilized for statistical analysis of the layer thickness of the 3D-printed samples.

## 3. Results and Discussion

### 3.1. Rheological Behavior and Printability of Al_2_O_3_ HM-Modified Pastes via 3D Printing

**Figure 3:**
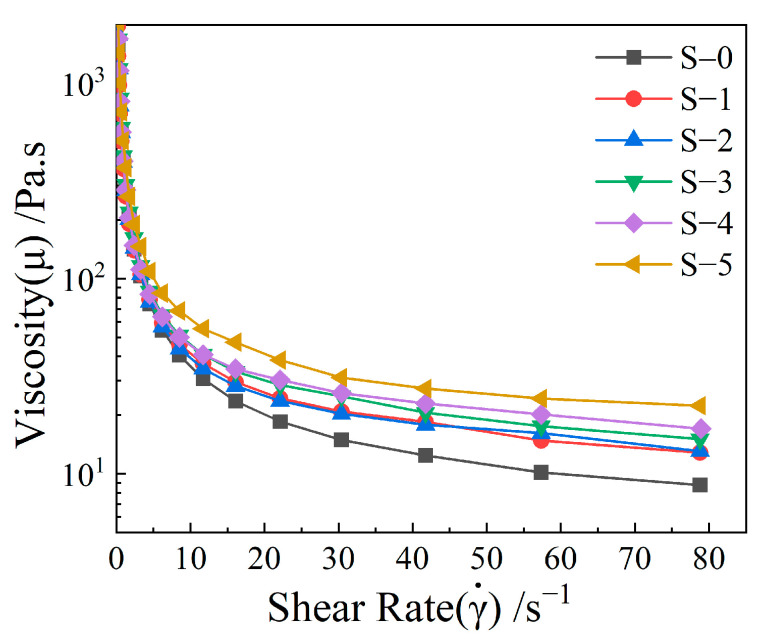
Rheological characteristic curves of pastes with different Al_2_O_3_ HM contents.

In this work, the as-prepared pastes were essentially a fluid with multiple dispersed phases. The deformation and flow of the pastes were determined by measuring the internal resistance under external extrusion force during the 3D printing process. Therefore, an evaluation of their rheological properties was required prior to ascertaining their printability. Figure 3 shows how a change in Al_2_O_3_ HM content generated corresponding fluctuations in the rheological properties of all pastes. The rheological behavior of all pastes could be divided into three distinct phases: (1) an initial phase characterized by a sharp decline in apparent viscosity as the shear rate increased from 0 to 10 s^−1^; (2) a transitional phase occurring between a shear rate from 10 to 65 s^−1^, where viscosity decreased at a slower rate; (3) a final stage, observed at a shear rate between 65 and 80 s^−1^, characterized by a less prominent viscosity change. Furthermore, the apparent viscosity of the ceramic pastes significantly increased with the addition of Al_2_O_3_ HM, consistent with previous findings [35]. These results indicated that the rheological properties of the ceramic pastes can be readily modified by varying the number of Al_2_O_3_ HM. Furthermore, under fixed Al_2_O_3_ HM content conditions, the modified pastes exhibited a substantial initial decrease, following a gradual decline, ultimately stabilizing as the shear rate increased. This indicates that the modified 3D printing pastes exhibited shear-thinning characteristics, as reported in previous studies [23,36].

**Figure 4:**
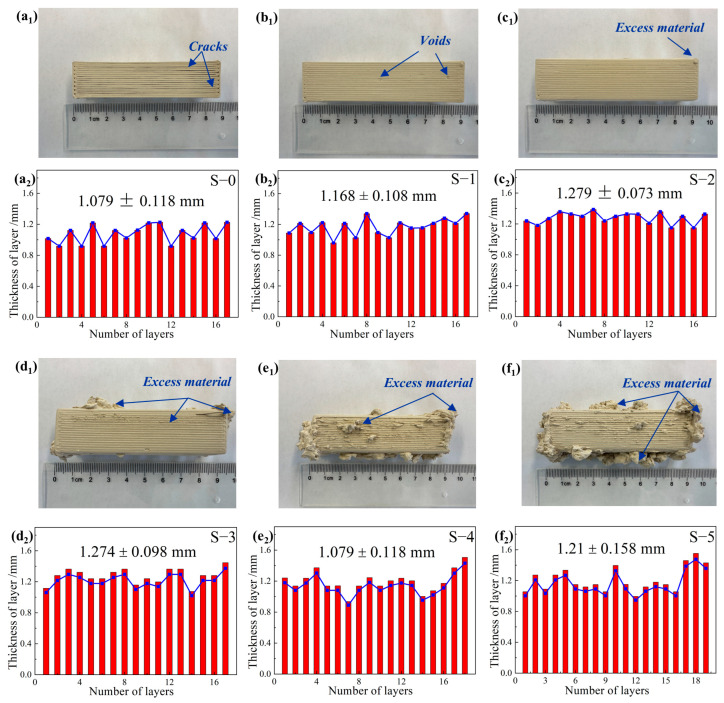
3D-printed samples using ceramic pastes with different Al_2_O_3_ HM contents and their corresponding layer thickness distribution: (**a_1_**,**a_2_**) 0 wt.%, (**b_1_**,**b_2_**) 3 wt.%, (**c_1_**,**c_2_**) 6 wt.%, (**d_1_**,**d_2_**) 9 wt.%, (**e_1_**,**e_2_**) 12 wt.%, and (**f_1_**,**f_2_**) 15 wt.%.

As is well known, the rheological behavior of ceramic pastes significantly impacts their printability for DIW 3D printing [37]. Clay samples printed using pastes modified with different Al_2_O_3_ HM contents are illustrated in Figure 4. The pristine S-0 sample showed an average layer thickness of 1.08 ± 0.12 mm, with minimal spacing between layers. The addition of 3 wt.% Al_2_O_3_ HM effectively mitigated interlayer spacing, resulting in an increase in the average thickness of each layer to 1.168 ± 0.11 mm. Moreover, a small number of void defects appeared (Figure 4b_1_). With an increase in Al_2_O_3_ HM content to 6 wt.% (S-2), the clay sample showed a smoother surface, with the layer thickness increasing to 1.28 ± 0.07 mm, and no noticeable spacing or voids were observed. However, it was noted that samples containing Al_2_O_3_ HM exceeding 6 wt.% (S-3, S-4, and S-5) exhibited a significant quantity of excess slurry adhered between the layers or edges of the clays. This led to unsatisfactory shape formation and decreased printability of the samples. Based on the rheological and 3D printability evaluation of the ceramic pastes, it can be concluded that all pastes were amenable to smooth extrusion. Among the pastes evaluated, sample S-2 exhibited the highest level of printability without noticeable spacing, voids, or excess material. This could be attributed to its moderate rheological behavior.

### 3.2. Microstructure of Fired 3D-Printed Ceramics

**Figure 5:**
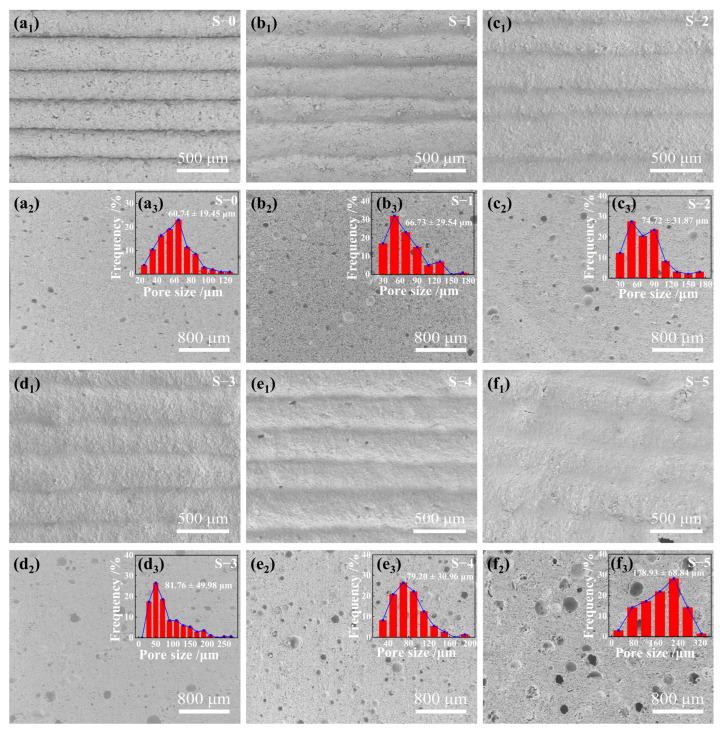
SEM images of Longquan celadon samples with different Al_2_O_3_ HM contents fired at 1280 °C (side view (**a_1_**–**f_1_**), cross-section (**a_2_**–**f_2_**), and pore size distribution (**a_3_**–**f_3_**)).

The as-prepared Al_2_O_3_ HM-modified 3D-printed samples were fired according to the temperature–time curve shown in Figure 2, after which the corresponding microstructure analysis was conducted. SEM images were captured for all the samples fired at 1250 °C, 1280 °C, and 1310 °C (the SEM images for the samples fired at 1250 °C and 1310 °C are shown in Figures S3 and S4). All these samples showed different microstructures at different temperatures. Figure 5 shows the samples fired at 1280 °C, which were significantly different in terms of the microstructure of the interlayers and micropores. The side-view SEM image in Figure 5a_1_ reveals interlayer gaps in S-0, which were effectively mitigated with increasing Al_2_O_3_ HM content (Figure 5b_1_–f_1_). The increase in Al_2_O_3_ HM content up to 15 wt.% led to a blurrier appearance of the interlayers and microstructural collapse between layers, consistent with the printability analysis results. Moreover, the cross-section SEM images revealed a substantial number of surface pores (Figure 5a_2_–f_2_). S-0 exhibited relatively fewer and smaller surface pores compared to other samples, with an average diameter of 60.74 ± 19.45 μm (Figure 5a_3_). In comparison, with the addition of 3 wt.% and 6 wt.% Al_2_O_3_ HM, the pore sizes increased to 66.73 ± 22.54 μm (Figure 5b_3_) and 74.72 ± 31.87 μm (Figure 5c_3_), respectively. Al_2_O_3_ HM content exceeding 6 wt.% led to the formation of larger surface pores (Figure 5d_2_–f_2_), potentially reducing the microstructural compactness and overall density, which, in turn, can lead to a decline in bending strength [35].

**Figure 6:**
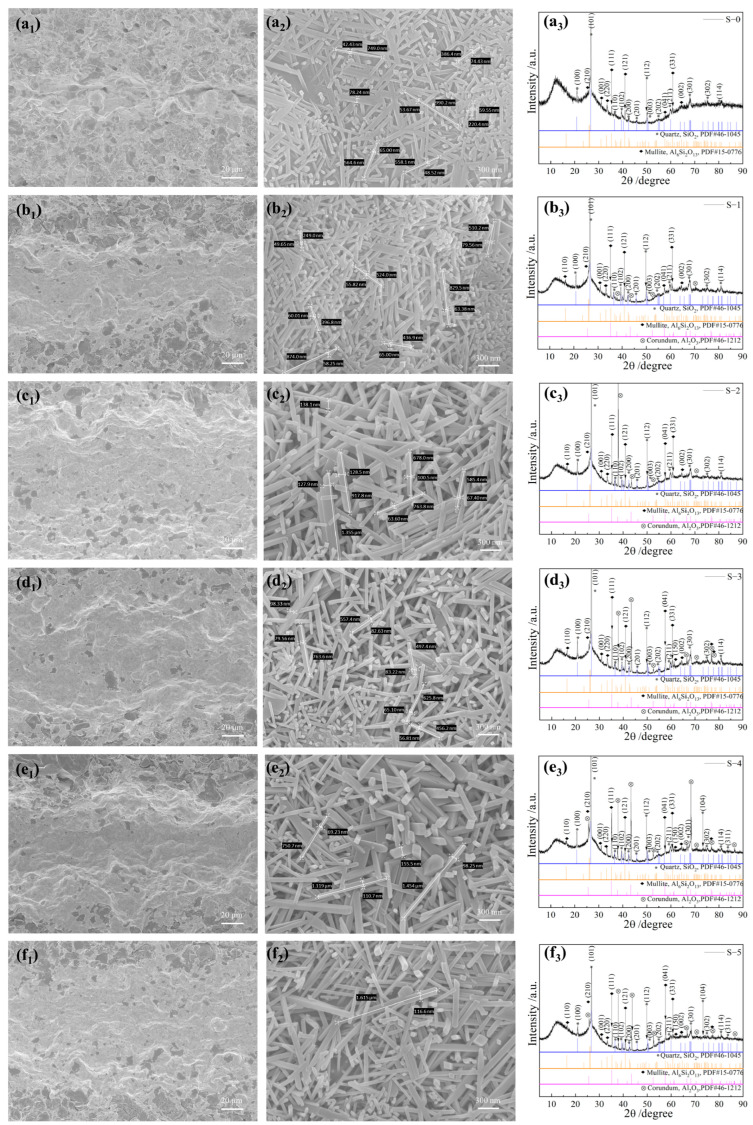
SEM images and XRD spectra of Longquan celadon samples with different Al_2_O_3_ HM contents fired at 1280 °C ((**a_1_**–**a_3_**) 0 wt.%, (**b_1_**–**b_3_**) 3 wt.%, (**c_1_**–**c_3_**) 6 wt.%, (**d_1_**–**d_3_**) 9 wt.%, (**e_1_**–**e_3_**) 12 wt.%, (**f_1_**–**f_3_**) 15 wt.%).

To further elucidate the intricate microstructural details of the Al_2_O_3_ HM-modified celadon ceramics, samples fired at 1280 °C underwent a 30 s treatment with 0.05 wt.% of hydrofluoric acid solution. This treatment effectively removed the glass phase from the ceramic surface, revealing numerous mullite whiskers. Data regarding the diameter and aspect ratio of the mullite whiskers are depicted in Figure 6 and summarized in Table S5. It is evident from Figure 6a_1_ that the distribution area of the mullite whiskers in the printing layer area was relatively smaller for samples with no Al_2_O_3_ HM addition. These mullite whiskers had diameters ranging from 42.43 to 78.24 μm and aspect ratios between 5.19 and 12.66 (seen in Figure 6a_2_). The corresponding phases were mainly composed of SiO_2_ (PDF # 46-1045) and mullite (PDF # 15-0776) (see Figure 6a_3_). When Al_2_O_3_ HM content increased from 3 wt.% to 9 wt.%, there was a significant expansion in the distribution area of mullite whiskers on the surface.

**Figure 7:**
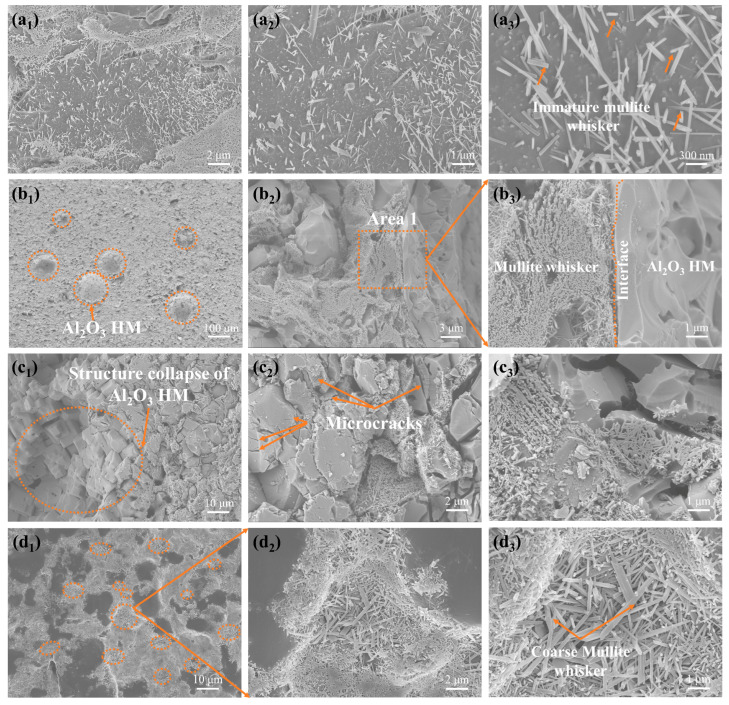
SEM images of the interface microstructure of the sample modified with 6 wt.% Al_2_O_3_ HM fired at 1250 °C (**a_1_**–**a_3_**), 1280 °C (**b_1_**–**b_3_**), and 1310 °C (**c_1_**–**c_3_**,**d_1_**–**d_3_**).

From the interlayer interface to both sides (see Figure 6b_1_–d_1_), there was minimal change observed in the diameter and aspect ratio of the mullite whiskers (see Figure 6b_2_–d_2_). The phases identified were composed of SiO_2_ (PDF # 46-1045), mullite (PDF # 15-0776), and Al_2_O_3_ (PDF # 46-1212) (see Figure 7b_3_–d_3_). With an increase in the content of Al_2_O_3_ HM to 12 wt.% and 15 wt.%, there was a slight reduction in the distribution area of the mullite whiskers in the printing layer (see Figure 6e_1_,f_1_). The diameter range of the mullite whiskers was 30.45~155.5 μm, with an aspect ratio ranging from 9.35 to 13.85 (see Figure 6e_2_,f_2_). There was no alteration in the phase composition (see Figure 6e_3_,f_3_). It is evident that an appropriate amount of Al_2_O_3_ HM content (6 wt.% and 9 wt.%) is beneficial in achieving the maximum distribution area of the mullite whisker structure, consistent with the improved bending strength discussed earlier.

The detailed SEM images in Figure 7a_1_–d_3_ reveal a significant correlation between firing temperature and the microstructure of the 3D-printed samples. As the firing temperature increased from 1250 °C to 1310 °C, a noticeable transformation occurred in the microstructure of the samples. At 1250 °C, immature mullite whiskers (arrows) were observed (see Figure 7a_1_–a_3_). At 1280 °C, a large amount of mullite whiskers were observed along the interface by enlarging the area 1 (square) of the image (see Figure 7b_1_–b_3_). In comparison, at 1310 °C, the structureal collapse (circle) of Al_2_O_3_ HM, microcracks (arrows) (see Figure 7c_1_–c_3_), and coarse mullite whiskers (arrows) (see Figure 7d_1_–d_3_) were observed, resulting in a decline in sample properties. Figure 7 indicates a significant presence of whiskers along the interface between the Al_2_O_3_ HM shell and the clay matrix (see Figure 7b_1_–b_3_). This observation was further confirmed by XRD spectroscopy, which showed that the observed whiskers along the interface belonged to the mullite structure (see Figure 7b_3_). Meanwhile, the core of Al_2_O_3_ HM exhibited no obvious whiskers and remained in a hollow structure, providing support to the clay in the form of a skeleton. This underscores the crucial role of Al_2_O_3_ HM in reducing the density of the 3D-printed samples while maintaining their bending strength. By controlling the concentration of Al_2_O_3_ HM, 3D-printed samples with properties equivalent to the Chinese Yuan Dynasty Longquan celadon restoration material can be achieved.

**Figure 8:**
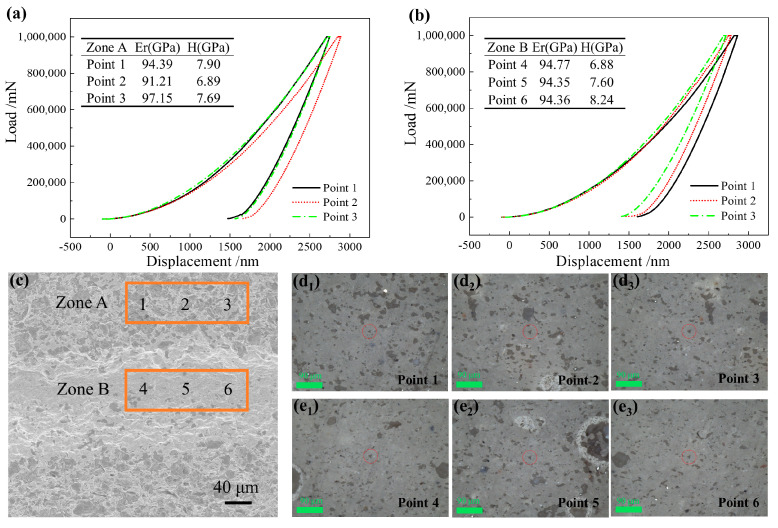
Displacement–load curves of Zone A (**a**) and Zone B (**b**) and micromechanical property data (see insert table) for the interlayer zone (**c**) and the impression in Zone A (**d_1_**–**d_3_**) and in Zone B (**e_1_**–**e_3_**).

Furthermore, the 6 wt.% Al_2_O_3_ HM-modified sample, which was fired at 1280 °C, played a crucial role in this experiment. It was used as an example to test the micromechanical properties (elastic modulus and hardness) of the interlayer zone of the 3D-printed sample, which were tested using a nanoindentation instrument [38,39]. A displacement–load curve and the micromechanical properties are shown in Figure 8a–c, and the impression images are presented in Figure 8d_1_–e_3_. From Figure 8a, it can be seen that the elastic modulus and hardness of the three points in zone A (Orange square) (see insert table data of zone A, Point 1–3) were not significantly different. An average elastic modulus of 94.25 GPa with a standard deviation of 2.43, as well as an average hardness of 7.49 GPa with a standard deviation of 0.44, were obtained. Similarly, the elastic modulus and hardness of three points in zone B (Orange square) (see insert table data of zone B, Point 4-5) were also not significantly different, with an average elastic modulus of 94.49 GPa with a standard deviation of 0.19, as well as an average hardness of 7.57 GPa with a standard deviation of 0.56. These consistent data underscore the fact that the elastic modulus and hardness of the 6 wt.% Al_2_O_3_ HM-modified sample prepared in this experiment are relatively close in the interlayer zone. This indicates that the overall mechanical properties of the interlayer zone of the sample from zone B to zone A are relatively uniform, mainly due to the relatively homogeneous microstructure in the interlayer zone.

### 3.3. Physical and Mechanical Properties of Fired 3D-Printed Ceramics

**Figure 9:**
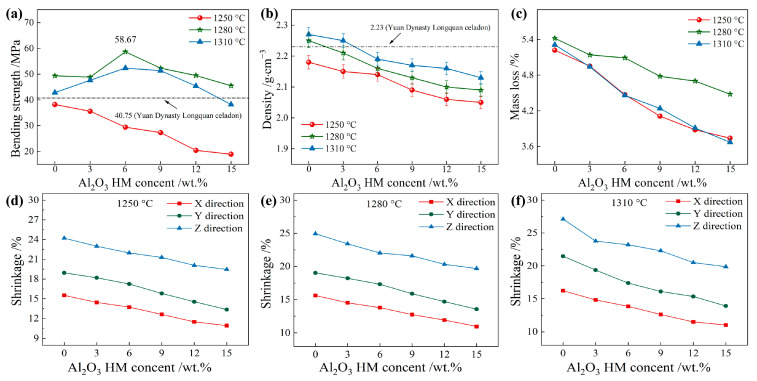
Physical and mechanical properties of Longquan celadon samples modified with different amounts of Al_2_O_3_ HM: (**a**) bending strength, (**b**) density, (**c**) mass loss, and (**d**–**f**) shrinkage at 1250~1310 °C.

Figure 9 presents crucial findings on the bending strength, density, shrinkage, and mass loss of samples fired at 1250 °C, 1280 °C, and 1310 °C with varying Al_2_O_3_ HM content, based on the foundation of previous research [40]. At 1250 °C, the bending strength gradually decreased with increasing Al_2_O_3_ HM content (Figure 9a), all falling below that of Yuan Dynasty Longquan celadon pottery (40.75 MPa). For samples fired over 1250 °C, the bending strength initially peaked, but then, gradually decreased as the Al_2_O_3_ HM concentration increased. It is also worth noting that samples fired at 1310 °C showed lower bending strength than those fired at 1280 °C, primarily due to the over-burning behavior caused by the higher firing temperature, leading to deteriorated mechanical performance [41,42,43,44].

In comparison with previous studies [45,46], all the fired 3D-printed ceramics in this experiment presented superior bending strength. Meanwhile, all samples exhibited decreasing density with increasing Al_2_O_3_ HM content (Figure 9b), with most samples possessing lower density than Yuan Dynasty Longquan celadon pottery (2.23 g∙cm^−3^). Density also decreased as firing temperature increased. The decrease in bending strength and density with Al_2_O_3_ HM content over 6 wt. % can be attributed to the notable increase in pore size and number. Similarly, all samples demonstrated reduced mass loss with increasing Al_2_O_3_ HM content (Figure 9c). Furthermore, as the content of hollow Al_2_O_3_ microspheres increased and the firing temperature increased, sample shrinkage of in the X, Y, and Z directions gradually decreased. Clearly, the shrinkage in the Z direction was higher than in the other two directions (Figure 9d–f). In short, a sample with optimal bending strength and moderate density can be obtained with 6 wt.% Al_2_O_3_ HM modification at a firing temperature of 1280 °C.

### 3.4. A Case Study of the Restoration of Chinese Yuan Dynasty Longquan Celadon

Following the analysis of the rheological, physical, and mechanical properties of the Al_2_O_3_ HM-modified pastes, the formulation containing 6 wt.% Al_2_O_3_ HM was selected for the 3D printing-based restoration of Chinese Yuan Dynasty Longquan celadon due to its optimal mechanical properties and printability. The firing temperature used for the restoration was 1280 °C as this provides a suitable bending strength and lower density comparable to those of excavated Yuan Dynasty Longquan celadon relics. Considering the shrinkage in the X, Y, and Z directions of the 6 wt.% Al_2_O_3_ HM-modified sample, appropriate margins were incorporated into the modeling sizes in all three directions, as outlined in Table 2, to guide the modeling size design.

**Figure 10:**
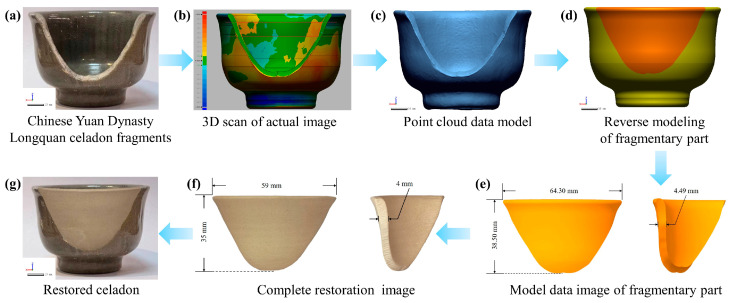
Schematic diagram of reverse modeling process of ancient ceramic fragments. (**a**) Chinese Yuan Dynasty Longquan celadon fragments, (**b**) 3D scan of actual image, (**c**) Point cloud data model, (**d**) Reverse modeling of fragmentary part, (**e**) Model data image of fragmentary part, (**f**) Complete restoration image, (**g**) Restored celadon.

Therefore, the modeling size was determined according to Table 2, and the shape of the missing fragment was reconstructed using the schematic diagram of the reverse modeling process illustrated in Figure 10. Central to this process was the utilization of a 3D laser scanner to construct a point cloud model [47], with the technical parameters detailed in Table S7. The model data of the fragmentary part were acquired through the reverse modeling process. Based on the shrinkage of the S-2 sample in the X, Y, and Z directions and the actual size of the missing parts, the modeling size was determined (see Table 2). Utilizing the 3D printer loaded with 6 wt.% Al_2_O_3_ HM-modified paste, the restoration of the Chinese Yuan Dynasty Longquan celadon fragmentary part was achieved (see Video S1). Moreover, the chemical composition of the restored missing part closely matched that of Yuan Dynasty Longquan celadon pottery (code: LQYD008), as confirmed by XRF testing (see Figure S5). Following a thermostability test of the 6 wt.% Al_2_O_3_ HM-modified missing part, it was observed that its surface remained free of cracks during the water exchange process, where the water temperature sharply decreased from 180 °C to 20 °C, thereby meeting the practical application requirements of Longquan celadon renovation materials.

## 4. Conclusions

In summary, utilizing a pseudo-classic clay imitating Chinese Yuan Dynasty Longquan celadon pottery as raw materials and incorporating hollow Al_2_O_3_ microspheres as modifiers, a series of 3D printing pastes with controllable rheological properties were prepared. Employing the DIW 3D printing technique, we fabricated the restored parts with reduced weight and enhanced mechanical properties. Increasing the Al_2_O_3_ HM content can enhance the apparent viscosity of the ceramic paste, giving it readily adjustable rheological properties. The 3D-printed samples achieved properties equivalent to those of the Chinese Yuan Dynasty Longquan celadon pottery fragmentary part. Specifically, when the content of Al_2_O_3_ HM was 6 wt.% in the modified paste, the firing temperature of the 3D-printed green body was 1280 °C, and the maximum bending strength of the fired 3D-printed ceramic reached 56.66 MPa with a relatively lower density of 2.16 g∙cm^−3^. By integrating DIW 3D printing technology with 3D laser scanning and 3D construction modeling techniques, along with Al_2_O_3_ HM-modified paste, successful restoration of the Chinese Yuan Dynasty Longquan celadon pottery fragmentary part (code: LQYD008) was achieved. This research offers a promising pathway for the damage-free restoration of cultural relics, harnessing the benefits of non-contact 3D printing while meeting the physical and aesthetic requirements for preserving ancient ceramics. Furthermore, it outlines a clear direction for future research, including the exploration of alternative materials for 3D printing pastes and the extension of this technique to diverse ceramic types.

## Figures and Tables

**Table 1:** Formulations of the Al_2_O_3_ HM-modified pastes.

Sample Name	Mass Fraction of Al_2_O_3_ HM (wt.%)	Pseudo-Classic Clay (g)	H_2_O (g)	HPMC (g)	Al_2_O_3_ HM (g)
S-0	0	2000	860	0.4	0
S-1	3	1940	860	0.4	60
S-2	6	1880	860	0.4	120
S-3	9	1820	860	0.4	180
S-4	12	1760	860	0.4	240
S-5	15	1700	860	0.4	300

**Table 2:** Dimensional design of Longquan celadon fragmentary part.

Items	X Direction	Y Direction	Z Direction
Shrinkage of S-2 sample/%	8.24	9.1	10.84
Actual size/mm	59	35	4
Modeling size/mm	64.30	38.5	4.49

## Data Availability

Data will be made available on request.

## Supplementary Materials

The following supporting information can be downloaded at: https://www.mdpi.com/article/10.3390/ma17071603/s1, Figure S1: (a) SEM image, (b) XRD pattern, and (c) particle size distribution of the pseudo-classic clay; Figure S2: (a–d) SEM image and EDS elemental mapping, (e) XRD pattern, (f) particle size distribution of Al_2_O_3_ HM; Figure S3: SEM images of Longquan celadon samples modified by different Al_2_O_3_ HM content fired at 1250 °C (side view (a_1_–f_1_) and cross-section (a_2_~f_2_)); Figure S4: SEM images of Longquan celadon samples modified by different Al_2_O_3_ HM content fired at 1310 °C (side view (a_1_–f_1_) and cross-section (a_2_~f_2_)); Figure S5: The restoration missing part images (a) and its composition by XRF testing (b,c); Table S1: The formula of the raw materials of pseudo-classic clay (wt.%); Table S2: The formula ingredient calculation process of pseudo-classic clay (wt.%); Table S3: The formula ingredient calculation process of pseudo-classic clay (wt.%); Table S4: Composition of the pseudo-classic clay and Chinese Yuan Dynasty Longquan celadon pottery (code: LQYD008) (wt.%); Table S5: Mullite whiskers diameter and aspect ratio of the modified Longquan Celadon samples; Table S6: Fracture toughness of the fired ceramics of Longquan celadon samples modified with different amount of Al_2_O_3_ HM at 1280 °C; Table S7: Scanning technical parameters of 3D laser scanner; Video S1: 3D printing process of the Chinese Yuan Dynasty Longquan celadon fragmentary part.
