# Supplementary material for: Processing, Microstructure, and Performance of Robocast Clay-Based Ceramics Incorporating Hollow Alumina Microspheres
Source: Materials (Basel). 2024 Mar 31;17(7):1603. doi: 10.3390/ma17071603 (PMC11012627; doi:10.3390/ma17071603)
Supplement: Supplementary file 1 [file materials-17-01603-s001.zip › materials-2899102-supplementary.pdf]

materials

MDPI

Article

Processing, Microstructure, and Performance of Robocast Clay-Based Ceramics Incorporating Hollow Alumina Microspheres

Yanfang Wu [1,2], Junjie Lan [3], Mingxuan Wu [2], Mingjun Wu [4], Li Tian [4], Hui Yang [1,3], Qijiang Li [5*], Yue Li [1,3*]

[1] School of Materials Science and Engineering, Zhejiang University, Hangzhou 310058, China; 11926036@zju.edu.cn (Y.F.W.); yanghui@zju.edu.cn (H.Y.)
[2] Chinese Celadon Institute, Lishui University, Lishui 323000, China; wumingxuan2023@gmail.com(M.X.W.)
[3] Wenzhou Research Institute, Zhejiang University, Wenzhou 325006, China; lanjj1127@126.com (J.J.L.)
[4] Longquan Celadon Museum, Longquan 323700, China; wumingjunlq@163.com (M.J.W.); emmett323700@gmail.com (L.T.)
[5] Research Center of Ancient Ceramic, Jingdezhen Ceramic University, Jingdezhen 333001, China
* Correspondence: authors: liqijiang@jcu.edu.cn; liyue8@zju.edu.cn

Abstract: The restoration of ancient ceramics has attracted widespread attention as it can reveal the overall appearance of ancient ceramics as well as the original information and artistic charm of cultural relics. However, traditional manual restoration is constrained due to its time-consuming nature and susceptibility to damaging ancient ceramics. Herein, a three-dimensional (3D) printing technique was employed to accurately restore Chinese Yuan Dynasty Longquan celadon using hollow Al_2O_3 microsphere-modified 3D printing paste. The results show that the hollow Al_2O_3 microsphere content plays a vital role in the printability, physical properties, and firing performance of the modified 3D printing paste. The printed green bodies show no noticeable spacing or voids under moderate rheological conditions. The as-prepared ceramic body modified with 6 wt.% hollow Al_2O_3 microspheres and fired at 1280 °C exhibits optimal bending strength of 56.66 MPa and a relatively low density of 2.16 g·cm^{-3}, as well as a relatively uniform longitudinal elastic modulus and hardness along the interlayer. This 3D printing technique based on hollow Al_2O_3 microsphere-modified paste presents a promising pathway for achieving non-contact and damage-free restoration of cultural relics.

Keywords: 3D printing; non-contact restoration; hollow Al_2O_3 microspheres; Longquan celadon; lightweight reinforcement

2. Materials and Methods

2.1. Preparation of Al_2O_3 HMs modified pastes

Table S1~S4 presented the preparation of a pseudo-classic clay. In order to successfully prepare a pseudo-classic clay with its composition consistent with that of Longquan celadon pottery (LQYD008), Huang tan porcelain clay, Zhu xiang porcelain clay, Zi jin clay, porcelain powder and potassium feldspar were used as the materials for imitation of Longquan celadon pottery, The formula for the pseudo-classic clay was shown in Table S1. Based on hierarchically satisfaction [1], the designed formula of pseudo-classic clay of Longquan celadon pottery was 40 wt.% Huang tan clay, 32 wt.% Zhu xiang clay, 20 wt.% Zi jin clay, 4 wt.% porcelain powder, and 4 wt.% potassium feldspar (see **Table S2** and **Table S3**) [2]. Then weighed the formula of the pseudo-classic clay, and added to a high-energy ball mill jar (ball to raw material weight ratio was 1:1). The dispersing medium was deionized water (deionized water to raw material weight ratio was 1:1.5). After milling at a speed of 400 r/min for 60 min, the as-prepared slurry was sieved through a 100

mesh sieve and dried at 80 °C. Eventually, the pseudo-classic clay was obtained. By XRF analysis, its composition was essentially consistent with that of LQYD008 (see **Table S4**).

Table S1. The formula of the raw materials of pseudo-classic clay (wt.%).

Raw material	SiO_2	Al_2O_3	CaO	MgO	K_2O	Na_2O	Fe_2O_3	TiO_2
Huang tan clay	70.46	21.14	0.07	0.49	5.98	1.35	0.47	0.02
Zhu xiang clay	69.61	23.33	0.06	0.41	5.40	0.45	0.71	0.02
Zi jin clay	56.25	32.15	0.06	1.25	4.29	0.52	5.10	0.38
Potassium feldspar	75.56	16.73	0.47	/	3.71	3.39	0.02	0.12
Porcelain powder	72.41	19.06	0.32	0.63	4.82	0.67	1.01	0.08

Table S2. The formula ingredient calculation process of pseudo-classic clay (wt.%).

Ingredient calculation	SiO_2	Al_2O_3	CaO	MgO	K_2O	Na_2O	Fe_2O_3	TiO_2
LQYD008	67.38	23.97	0.05	0.83	5.71	0.31	1.64	0.11
Zhu xiang clay 32%	22.28	7.47	0.02	0.13	1.73	0.14	0.23	0.01
residual	45.10	16.50	0.03	0.70	3.98	0.17	1.41	0.10
Huang tan clay 40%	28.18	8.46	0.03	0.20	2.39	0.54	0.19	0.01
residual	16.92	8.05	0.00	0.50	1.59	-0.37	1.22	0.10
Zi jin clay 20%	11.25	6.43	0.01	0.19	0.86	0.10	1.02	0.08
residual	5.67	1.62	-0.01	0.31	0.73	-0.48	0.20	0.02
Porcelain powder 4%	2.93	0.77	0.01	0.03	0.19	0.03	0.08	0.01
residual	2.75	0.85	-0.02	0.29	0.54	-0.50	0.12	0.01
Potassium feldspar 4%	3.02	0.67	0.02	0.00	0.15	0.14	0.00	0.00
residual	-0.28	0.18	-0.04	0.29	0.39	-0.64	0.12	0.01

Table S3. The formula ingredient calculation process of pseudo-classic clay (wt.%).

Huang tan clay	Zhu xiang clay	Zi jin clay	Porcelain powder	Potassium feldspar
40	32	20	4	4

Table S4. Composition of the pseudo-classic clay and Chinese Yuan Dynasty Longquan celadon pottery (code: LQYD008) (wt.%).

Sample	SiO_2	Al_2O_3	CaO	MgO	K_2O	Na_2O	Fe_2O_3	TiO_2

LQYD008	67.38	23.97	0.05	0.83	5.71	0.31	1.64	0.11
Pseudo-classic clay	67.10	24.15	0.01	1.12	6.10	0.13	1.76	0.12

Figure S1 presented the morphologies, phase and size distribution of the pseudo-classic clay. SEM micrograph (Figure S1a) revealed the pseudo-classic clay was irregular particle. XRD pattern (Figure S1b) showed that the material was mainly composed of quartz phase (SiO_2) and potassium feldspar phase ($KAlSi_3O_8$). Particle size (Figure S1c) analysis indicated a wide particle size distribution ranging from 1~100 μm with D_{50} particle diameter at 8.9 μm.

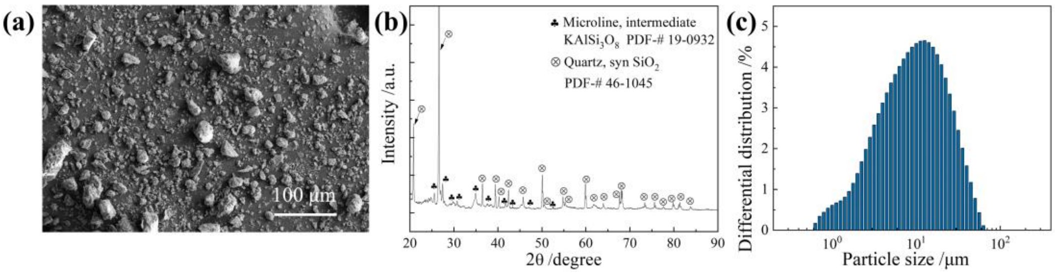

Figure S1. (a) SEM image, (b) XRD pattern, and particle size distribution of the pseudo-classic clay (c).

Figure S2 presented morphologies, phase and size distribution of Al_2O_3 HM. SEM image in Figure S2a indicated that the Al_2O_3 HM were spherical in shape and nearly monodisperse. The corresponding EDS elemental analysis (Figure S2b~d) also revealed uniform distribution of Al and O element. Some surface defects were visible on the surface of Al_2O_3 HM, which presented the evident existence of inside hollow structure. XRD pattern of Al_2O_3 HM in Figure S2e indicated a single crystalline corundum phase of the microspheres with hexagonal crystal structure that belonged to space group R-2c. Particle size analysis in Figure S2f indicated a relatively narrow size distribution of the microspheres with average diameter of 133 μm.

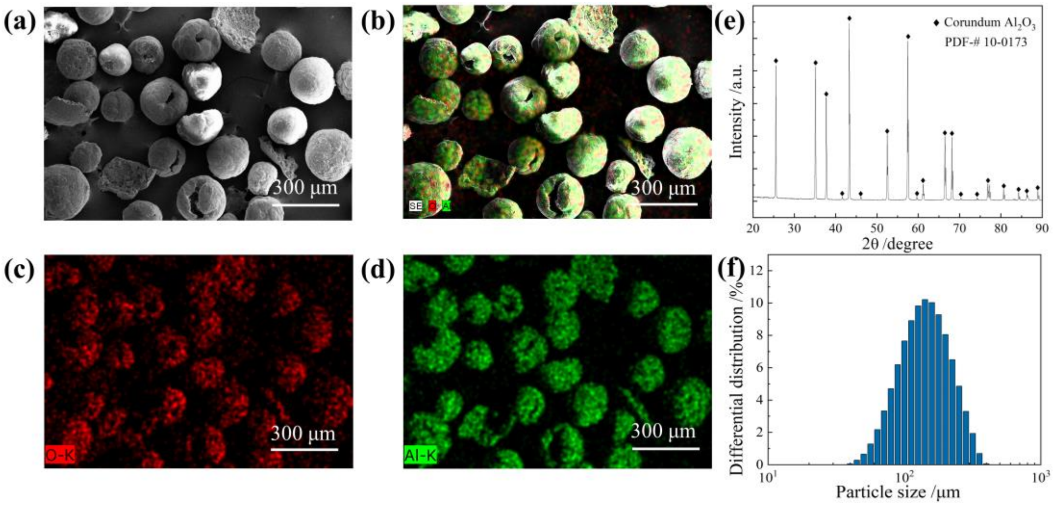

Figure S2. (a~d) SEM image and EDS elemental mapping, (e) XRD pattern, (f) particle size distribution of Al_2O_3 HM.

Figure S3~S4 presented the SEM images of Longquan celadon samples modified by

different Al_2O_3 HM content fired at ((a_1~f_2) 1250 °C and (a_1~f_2) 1310 °C. By observing the SEM images in Figure S3, there had clear presence of interlayer gaps on the samples with Al_2O_3 HM content less than 6 wt.% when fired at 1250 °C, and the interlayers of the samples with Al_2O_3 HM content over 9 wt.% became more blurred and interlayer microstructural collapse. Figure S4 presented interlayer gaps with Al_2O_3 HM content of 0 wt.% when fired at 1310 °C, while, similar microstructure characteristics were also observed for the samples with Al_2O_3 HM content over 3 wt.%.

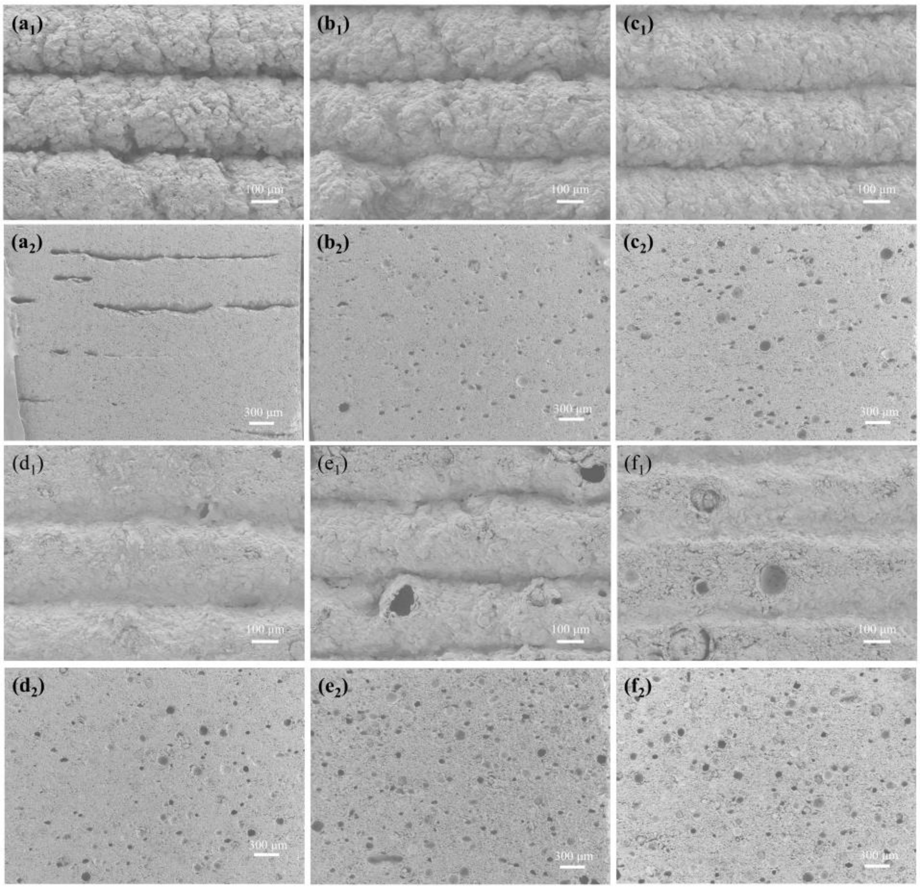

Figure S3. SEM images of Longquan celadon samples modified by different Al_2O_3 HM content fired at (1250 °C (side view (a_1~f_1) and cross-section (a_2~f_2)).

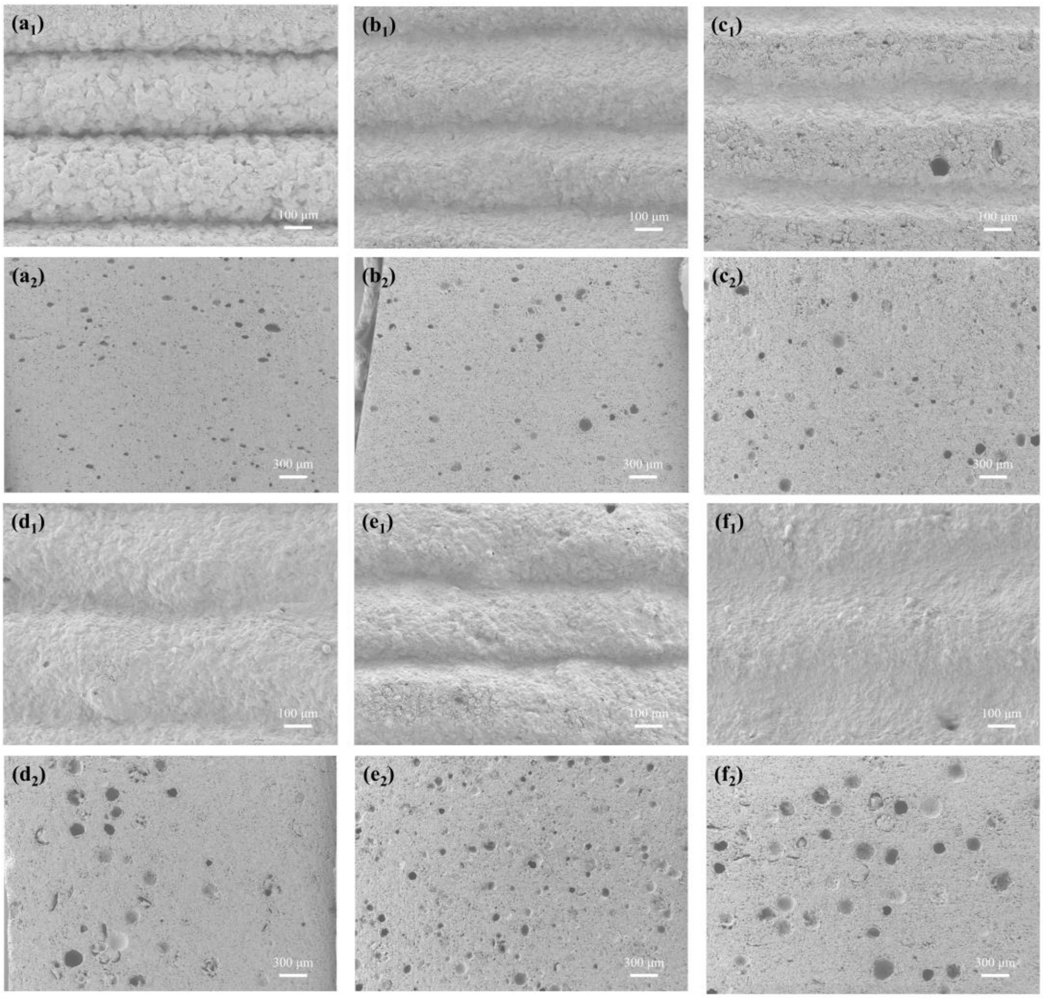

Figure S4. SEM images of Longquan Celadon samples modified by different Al_2O_3 HM content fired at (1310 °C (side view (a_1~f_1) and cross-section (a_2~f_2)).

Figure S5. presented the restoration missing part images and its composition by XRF testing. Through XRF analysis, the restoration missing part is mainly composed of 0.53% Na_2O, 1.08% MgO, 24.86% Al_2O_3, 66.51% SiO_2, 4.78% K_2O, 0.06% CaO, 0.06% TiO_2, 1.11% Fe_2O_3, and balance 1.01%. Thus, its composition of the restoration missing part is comparatively accordant with that of Chinese Yuan Dynasty Longquan celadon pottery (code: LQYD008) shown in Figure S5c.

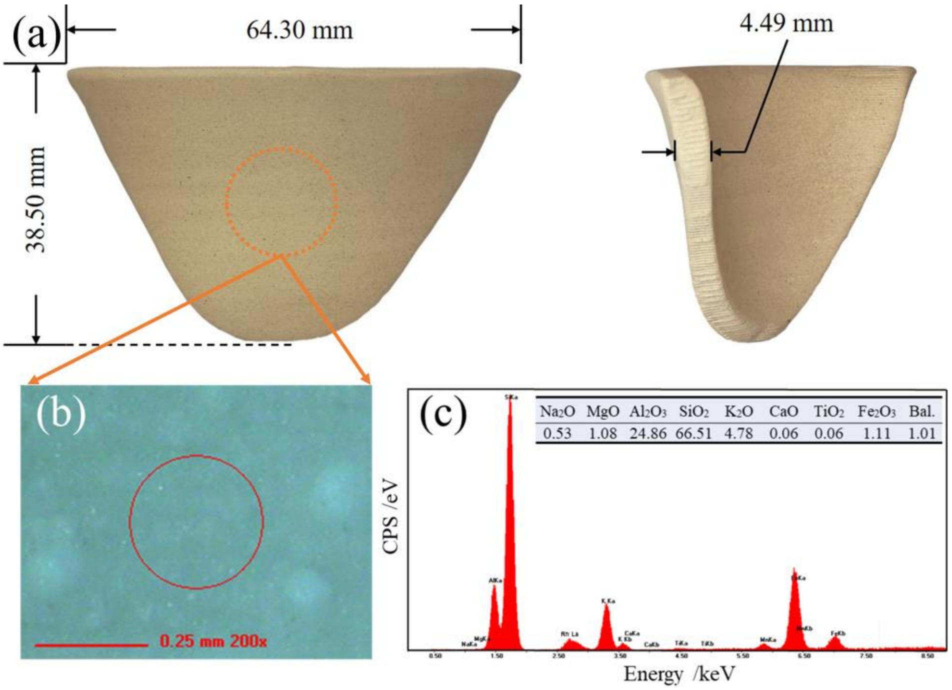

Figure S5. The restoration missing part images(a) and its composition by XRF testing (b~c).

The data on the diameter and aspect ratio of mullite whiskers shown in Figure 6 have been organized in a table S5.

Table S5. Mullite whiskers diameter and aspect ratio of the modified Longquan Celadon samples.

Sample name	Mass fraction of Al_2O_3 HMs (wt.%)	mullite whiskers diameter (nm)	aspect ratio
S-0	0	42.43 ~ 78.24	3.70 ~ 18.45
S-1	3	49.65 ~ 79.56	5.02 ~ 15.00
S-2	6	63.60 ~ 138.10	6.75 ~ 12.01
S-3	9	56.81 ~ 98.33	5.98 ~ 9.61
S-4	12	69.23 ~ 155.50	10.11 ~ 14.80
S-5	15	116.60	13.85

Meanwhile, taking 6 wt.% Al_2O_3 HM modified sample fired at 1280 ℃ as an example, fracture toughness of the printed ceramics was also investigated. Fracture toughness of the fired ceramics was evaluated by integrating the stress-strain curve of the bending strength test. Table S6. presented the fracture toughness of the printed ceramics. It can be seen that with the increase of mass fraction of Al_2O_3 HM, the fracture toughness first increased peaking at 6 wt.% Al_2O_3 HMs (34.56 MPa·$m^{1/2}$), and later gradually declined to 22.41%.

Table S6. Fracture toughness and thermostability of the fired ceramics of Longquan celadon samples modified with different amount of Al_2O_3 HMs at 1280 ℃.

Sample name	Mass fraction of Al_2O_3 HM (wt.%)	Fracture toughness /MPa·$m^{1/2}$
S-0	0	21.38
S-1	3	24.79
S-2	6	34.56
S-3	9	31.15
S-4	12	26.35

S-5	15	22.41

Table S7. Scanning technical parameters of 3D laser scanner.

Items	Properties
Weight	840 g
Dimensions(L×W×H)	298 mm×103.5 mm×74.5 mm
Single shot accuracy	0.01 mm
Volume precision	0.02 mm+0.015 mm/m
Resolution	0.01 mm
Scan speed	2100000 scan/s
Maximum scanning area	600 mm×550 mm
Working distance	175 mm
Scan depth	510 mm
Light source	5-parallel blue laser lines

Formula S1 presented the calculation method for mass loss. Mass loss was determined by the following test process: the as-prepared samples were dried at 110 °C ± 5 °C for 5h, put them into a dryer to cool to room temperature, and weighed them as G_0 (±0.01g); Burned the blank sample at high temperature, dried it at 110 °C ± 5 °C for 5 h, and then weighed it as G_1 (±0.01 g) by the following **Formula S1**.

$$S = \frac{G_1 - G_0}{G_0} \times 100\% \qquad \text{(S1)}$$

In which, S- mass loss, %;

G_0- mass of dried samples, g;

G_1- mass of sintered samples, g.

Formula S2 presented the calculation method for density. Density was determined by Archimedes method. Firstly, measured the temperature of distilled water and checked the table to obtain the density of water at the corresponding temperature. After adjusting and clearing, weighed the weight of the sample in the air on the balance, then put the sample into distilled water to weigh the corresponding weight. And then the corresponding density values were calculated by the following **Formula S2**.

$$\rho = \frac{m_0 \rho_0}{m_0 - m_1} \times 100\% \qquad \text{(S2)}$$

In which, ρ- density of the sample, $g \cdot cm^{-3}$;

ρ_0- density of the distilled water at a certain temperature, $g \cdot cm^{-3}$;

m_0- mass in air, g;

m_1- mass in distilled water, g.

Author Contributions: Conceptualization, Y.W. and H.Y.; methodology, Y.W. and Y.L.; software, M.W.; validation, J.L. and Q.L.; formal analysis, J.L.; investigation, J.L.; resources, M.W. and L.T.; data curation, J.L.; writing—original draft preparation, Y.W.; writing—review and editing, Y.L. and Q.L.; visualization, M.W.; supervision, H.Y.; project administration, Y.L. and Q.L.; funding acquisition, H.Y. All authors have read and agreed to the published version of the manuscript.

Funding: This research was received by the National Natural Science Foundation of China (grant number 12171217) and Key Laboratory of Traditional Heated-form Craft Technology and Digital Design (China Academy Art), Ministry of Culture and Tourism (grant number TK202112310)

Institutional Review Board Statement: Not applicable.

Informed Consent Statement: Not applicable.

Data Availability Statement: Data will be made available on request.

Conflicts of Interest: The authors declare no conflicts of interest.

References

1. Ma, T.C. *Ceramic Technology*, 2nd ed.; China Light Industry Press: Beijing, China, 2013; pp. 120–125. (In Chinese)
2. Wang, J.H. Simplified calculation of chemical composition satisfy method in ceramic formula calculation. *Chin. Ceram.* **1994**, *5*, 17–21. (In Chinese)

Disclaimer/Publisher's Note: The statements, opinions and data contained in all publications are solely those of the individual author(s) and contributor(s) and not of MDPI and/or the editor(s). MDPI and/or the editor(s) disclaim responsibility for any injury to people or property resulting from any ideas, methods, instructions or products referred to in the content.
